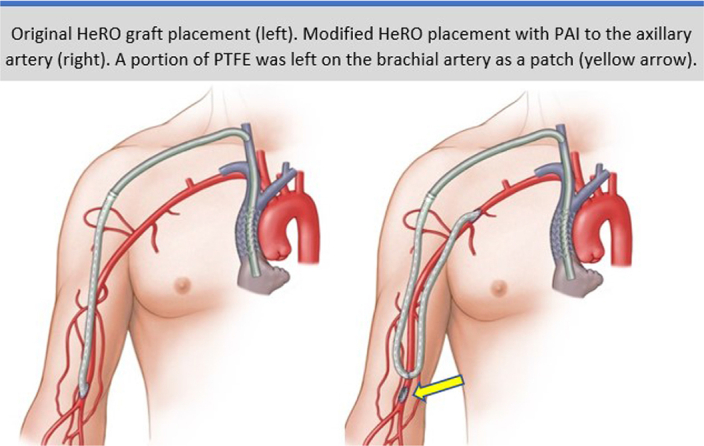# Modified proximalization of arterial inflow using a hemodialysis reliable outflow graft in a patient with vascular access failure

**DOI:** 10.1016/j.jvscit.2022.11.003

**Published:** 2022-11-15

**Authors:** Sebastian Cifuentes, Armin Tabiei, Randall R. DeMartino

**Affiliations:** Division of Vascular and Endovascular Surgery, Mayo Clinic, Rochester, MN

A 63-year-old man with end-stage renal disease and multiple failed vascular accesses (VAs) had been referred for creation of permanent dialysis access. He provided written informed consent for the report of his case details and imaging studies. His medical history included a failed kidney transplantation, aortic valve replacement, severe coronary artery disease requiring two coronary artery bypass grafts, and hypertension. A previous right groin and upper extremity VA had been complicated by chronic deep and central vein thrombosis, requiring stenting of the right innominate and superior vena cava (SVC) due to SVC syndrome. The patient was receiving chronic anticoagulation therapy and hemodialysis via a translumbar catheter. He had initially been offered a hemodialysis reliable outflow (HeRO) graft as the best option for permanent VA. Ultrasound-guided access to the right internal jugular vein was obtained, and the previously stented SVC was balloon dilated to maximize the lumen diameter (*A*). The venous outflow component of the HeRO graft was placed at the right atrial junction. After tunneling, the proximal, 6-mm polytetrafluoroethylene (PTFE) component was sewn end-to-side to the brachial artery above the antecubital crease (*B*). After 1 month, the patient had developed severe right hand pain during dialysis and ulceration of the third finger (*C*), findings consistent with arteriovenous steal. Distal revascularization with interval ligation (DRIL) was planned, but the saphenous vein was not adequate. Thus, proximalization of arterial inflow (PAI) was considered the best surgical option to preserve the HeRO graft access. PAI has been described as a suitable alternative to DRIL that will maintain the natural arterial pathway.^1^ A 6-mm PTFE graft was anastomosed end-to-side to the axillary artery and tunneled down to the brachial artery. The previous PTFE graft was disconnected from the brachial artery and sewn end-to-end to the new PTFE graft, creating a loop (*D*/Cover). After 1 month, the pain and finger wound had resolved, and dialysis was started through the HeRO graft. Ultrasound showed normal flow and patency after 3 months. The HeRO graft is an acceptable option for patients with complex dialysis requirements.^2^ However, dialysis access-induced ischemia can be a severe complication.^1^ For patients for whom DRIL is not an option, PAI using the HeRO graft is an innovative and effective alternative for achieving graft preservation and symptom resolution.

**Figure:**
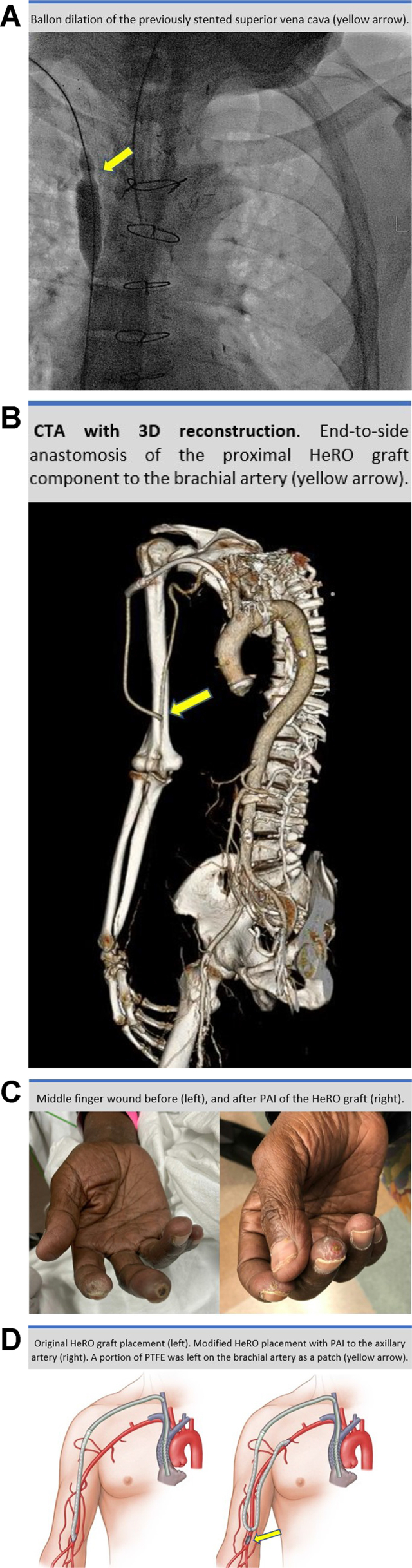

## Figures and Tables

**Cover Image:**